# Resident Burnout, Wellness, Professional Development, and Engagement Before and After New Training Schedule Implementation

**DOI:** 10.1001/jamanetworkopen.2024.0037

**Published:** 2024-02-28

**Authors:** Daniel Heppe, Amiran Baduashvili, Julia E. Limes, Katie Suddarth, Adrienne Mann, Emily Gottenborg, Yasmin Sacro, Lisa Davis, Karen Chacko, Geoffrey Connors

**Affiliations:** 1 University of Colorado Internal Medicine Residency, Department of Hospital Medicine, University of Colorado School of Medicine, Aurora; 2 Division of Hospital Medicine, University of Colorado School of Medicine, Aurora; 3 Division of Hospital Medicine, University of Colorado, Aurora; 4 Department of Medicine, University of Colorado School of Medicine, Aurora; 5 Department of General Internal Medicine, University of Colorado School of Medicine, Aurora; 6 Division of Rheumatology, Denver Health and Hospital Authority, Denver, Colorado; 7 Internal Medicine Training Program, University of Colorado School of Medicine, Aurora; 8 Internal Medicine Residency Program, Pulmonary and Critical Care Medicine, University of Colorado School of Medicine, Aurora

## Abstract

**Importance:**

Burnout is a work-related syndrome of depersonalization (DP), emotional exhaustion (EE), and low personal achievement (PA) that is prevalent among internal medicine resident trainees. Prior interventions have had modest effects on resident burnout. The association of a new 4 + 4 block schedule (4 inpatient weeks plus 4 outpatient weeks) with resident burnout has not previously been evaluated.

**Objective:**

To evaluate the association of a 4 + 4 block schedule, compared with a 4 + 1 schedule, with burnout, wellness, and self-reported professional engagement and clinical preparedness among resident physicians.

**Design, Setting, and Participants:**

This nonrandomized preintervention and postintervention survey study was conducted in a single academic-based internal medicine residency program from June 2019 to June 2021. The study included residents in the categorical, hospitalist, and primary care tracks in postgraduate years 1 and 2 (PGY1 and PGY2). Data analysis was conducted from October to December 2022.

**Intervention:**

In the 4 + 4 structure, resident schedules alternated between 4-week inpatient call-based rotations and 4-week ambulatory non–call-based rotations.

**Main Outcomes and Measures:**

The primary outcome was burnout, assessed using the Maslach Burnout Inventory subcategories of EE (range, 0-54), DP (range, 0-30), and PA (range, 0-48), adjusted for sex and PGY. Secondary outcomes included In-Training Examination (ITE) scores and a questionnaire on professional, educational, and health outcomes. Multivariable logistic regression was used to assess the primary outcome, 1-way analysis of variance was used to compare ITE percentiles, and a Bonferroni-adjusted Kruskal Wallis test was used for the remaining secondary outcomes. The findings were reexamined with several sensitivity analyses, and Cohen’s *D* was used to estimate standardized mean differences (SMDs).

**Results:**

Of the 313 eligible residents, 216 completed the surveys. A total of 107 respondents (49.5%) were women and 109 (50.5%) were men; 119 (55.1%) were PGY1 residents. The survey response rates were 78.0% (85 of 109) in the preintervention cohort and 60.6% (63 of 104) and 68.0% (68 of 100) in the 2 postintervention cohorts. The PGY1 residents had higher response rates than the PGY2 residents (119 of 152 [78.2%] vs 97 of 161 [60.2%]; *P* < .001). Adjusted EE scores (mean difference [MD], −6.78 [95% CI, −9.24 to −4.32]) and adjusted DP scores (MD, −3.81 [95% CI, −5.29 to −2.34]) were lower in the combined postintervention cohort. The change in PA scores was not statistically significant (MD, 1.4 [95% CI, −0.49 to 3.29]). Of the 15 items exploring professional, educational, and health outcomes, a large positive association was observed for 11 items (SMDs >1.0). No statistically significant change in ITE percentile ranks was noted.

**Conclusions and Relevance:**

In this survey study of internal medicine resident physicians, a positive association was observed between a 4 + 4 block training schedule and internal medicine resident burnout scores and improved self-reported professional, educational, and health outcomes. These results suggest that specific 4 + 4 block combinations may better improve resident burnout than a 4 + 1 combination used previously.

## Key Points

**Question:**

What is the association between a 4 + 4 block schedule (4 inpatient weeks plus 4 outpatient weeks) and resident wellness, burnout, self-reported professional engagement, and clinical preparedness?

**Findings:**

In this survey study with 216 nonrandomized respondents, marked improvements were observed in emotional exhaustion and depersonalization scores on the Maslach Burnout Inventory among internal medicine residency trainees after implementation of a 4 + 4 block schedule compared with the preintervention 4 + 1 schedule. There was no change in scores for personal achievement.

**Meaning:**

These findings suggest that 4 + 4 block scheduling, compared with 4 + 1 scheduling, may be associated with substantial improvement in resident burnout.

## Teaser

This survey study examines the association of block scheduling with burnout, wellness, professional engagement, and clinical preparedness among internal medicine residents.

## Introduction

Burnout is a work-related syndrome of depersonalization (DP), emotional exhaustion (EE), and low personal achievement (PA).^1^ Fatigue and moral distress among physicians, which are often associated with burnout, have substantial consequences for patient safety and career satisfaction. Among resident and practicing physicians, burnout is associated with self-perceived poor performance at work, self-reported errors, career disengagement, anxiety, depression, and intent to leave the profession.^2,3,4,5,6,7^ The postgraduate training period is a particularly stressful time for physicians.^8^ Before the COVID-19 pandemic, up to 75% of internal medicine residents showed symptoms of burnout.^9^ The pandemic likely worsened levels of burnout among resident trainees; in one study, 81% of internal medicine residents felt that the pandemic increased their level of burnout.^10^

Structural changes, wellness activities, mental health support initiatives, coaching, and adequate sleep have been suggested as mechanisms to address burnout among resident physicians.^7,11,12^ Duty-hour policies introduced in 2011 by the Accreditation Council for Graduate Medical Education have been associated with improvements in the EE and DP domains of the Maslach Burnout Inventory (MBI), a self-test for burnout.^13,14^ However, given the high prevalence of burnout among residents, additional interventions and innovations are needed to address burnout and foster wellness. These interventions and innovations can include improving the learning climate, increasing connectedness, and striving for joy in medicine and life balance during training, which can lead to thriving in residency.^15^ Furthermore, training schedules that create protected time for residents to engage in activities such as scholarship and quality improvement are needed, because these activities promote professional development and engage residents in mentorship relationships that can have long-lasting benefits.^16,17^

Block scheduling was developed as a mechanism to decrease the conflict between inpatient and outpatient responsibilities inherent to traditional resident training models and to increase the emphasis on training in the ambulatory setting.^18^ Studies have demonstrated that block scheduling may improve resident satisfaction with ambulatory training, decrease the conflict between inpatient and outpatient responsibilities, and improve sleep.^19,20^ In a 2015 survey, internal medicine residency program directors reported that block scheduling reduced resident stress and improved resident satisfaction and their ability to focus on their current rotation.^21^ However, the impact of block scheduling on career development and engagement is unknown, and its association with resident burnout has not been directly examined.

This study aimed to evaluate the association of a 4 + 4 block schedule (4 inpatient weeks plus 4 outpatient weeks), compared with a prior 4 + 1 block schedule (4 inpatient weeks plus 1 outpatient week), with resident wellness, burnout, and self-reported professional engagement and clinical preparedness. We hypothesized that this intervention would improve resident burnout, wellness, and professional engagement without reducing medical knowledge, as assessed by In-Training Examinations (ITE) scores or perceived clinical readiness.

## Methods

### Design, Setting, and Participants

This nonrandomized preintervention and postintervention survey study was conducted at a single academic internal medicine residency program (IMRP) involving postgraduate year 1 and 2 (PGY1 and PGY2) residents. The IMRP sites included a university, a US Veterans Administration hospital, and a city/county hospital. The Colorado Multiple Institutional Review Board reviewed and approved the study protocol; we provided residents with a consent form but a signature was not required to provide informed consent. Beginning with academic year 2019-2020, the IMRP implemented a new block scheduling system. Residents were invited to voluntarily complete surveys assessing burnout, educational outcomes, and quality of life both before and after this change. The surveys were anonymized, collecting only PGY status and sex. The study followed the Consensus-Based Checklist for Reporting of Survey Studies (CROSS) reporting guideline.

### Intervention

In July 2019, the IMRP launched a new rotation structure that was implemented throughout the program. Before the intervention, the rotation structure followed a 4 + 1 model, in which residents alternated between 4 inpatient weeks and 1 outpatient week. The intervention introduced a 4 + 4 model, in which resident schedules alternated between 4-week inpatient call-based and 4-week ambulatory non–call-based rotations. A detailed description of the rotation schedule is provided in the eAppendix in Supplement 1.

### Survey Instruments and Outcomes

The primary outcome was burnout, which was assessed using the MBI–Human Services Survey for Medical Personnel (MBI-HSS[MP])^1^ and encompassed 22 items across 3 subscales: EE, DP, and PA. The respondents rated each item on a 7-point Likert scale based on how frequently they experienced the described job-related feeling, ranging from 0 (never) to 6 (every day). We categorized subscales into levels of concern as follows: EE (range, 0-54): low (0-16), moderate (17-26), and high (27-54); DP (range, 0-30): low (0-6), moderate (7-12), and high (13-30); and PA (range, 0-48): high (0-31), moderate (32-38), and low (39-48).^22^ High EE and DP but low PA scores indicated greater burnout concern.

We assessed several secondary outcomes using a 15-item questionnaire utilized by the iCOMPARE study.^23^ This questionnaire was coadministered with the MBI-HSS(MP) to evaluate participants’ self-perceived impact of the rotation structure with various outcomes, including the ability to acquire clinical skills, access to educational and scholarly opportunities, job satisfaction, and health. Each item was scored on a 5-point Likert scale ranging from 1 (significantly negative) to 5 (significantly positive). The full survey instrument is provided in eAppendix 2 in Supplement 1.

As a surrogate of medical knowledge acquisition, we analyzed the ITE percentile ranks for PGY1 and PGY2 residents. We included 6 years of ITE scores: 2 years preceding and 4 years following the intervention. Because the surveys were anonymous, we could not exclude the ITE percentile ranks for the residents who opted out of the survey.

### Survey Administration and Participants

We administered the surveys at baseline (within 3 months before the intervention, June 2019) and at 1 year (June 2020) and 2 years (June 2021) after the intervention. The first survey was administered in person, whereas the subsequent 2 were virtual due to the COVID-19 pandemic. All categorical, hospitalist, and primary care track PGY1 and PGY2 internal medicine residents were eligible. We excluded preliminary PGY1, medicine-pediatrics, and PGY3 residents because they would be unavailable to complete the follow-up survey a year later. The 2020 cohort overlapped with both the 2019 and 2021 cohorts. Some PGY1 residents surveyed in 2020 likely participated in 2021 as PGY2 residents, and some PGY2 residents surveyed in 2020 likely participated in 2019 as PGY1 residents. The PGY3 exclusion helped maintain sample independence between 2019 and 2021.

### Statistical Analysis

Due to anonymity, individual tracking across 3 years was not possible, preventing paired analyses. Each cohort was treated as an independent sample. The unadjusted MBI-HSS(MP) subscale scores had skewed distribution and are presented using medians and IQRs. Differences in the overall survey response rates by PGY and sex were compared using a χ^2^ test. For the primary analysis, we evaluated the change in MBI-HSS(MP) subscale scores before and after the intervention (combined 2020 and 2021), adjusted for PGY and sex, using multivariable linear regression. Since the subscale scores were not normally distributed, we conducted a sensitivity analysis using a nonparametric Kruskal-Wallis (KW) test adjusted for ties, which treated 3 cohorts separately.^24^ The significance level was Bonferroni adjusted for multiple hypotheses (3 subscales) and set at *P* < .017. Whenever the KW test showed significance, the Dunn test enabled pairwise comparisons across the 3 cohorts,^25^ which separately compared the preintervention cohort to the postintervention cohorts with a partial resident overlap (2020) and fully independent sample (2021).

To preserve all responses, we did not dichotomize the Likert scale responses for secondary outcomes. Instead, we employed the KW test with Bonferroni correction (*P* < .003) for all 15 questions across 3 surveyed years. We performed the Dunn test for each statistically significant question. Additionally, we used Cohen’s *D* to estimate standardized mean differences (SMDs) for preintervention and pooled postintervention survey scores. Standardized mean differences of 0.2, 0.5, and 0.8 signify small, moderate, and large effect sizes, respectively.^26,27^

The ITE percentile ranks across 6 academic year cohorts were summarized using means with SDs and medians with IQRs. We used 1-way analysis of variance to assess whether the ITE ranks from any cohort differed statistically from the rest and the repeated analysis with KW test given skewed distribution. Next, we divided ITE ranks into the preintervention and postintervention groups and compared postintervention ranks to the preintervention mean using a 1-sample *t* test due to nonindependence. We repeated the analysis using the Wilcoxon rank-sum test. Finally, we used multivariable linear regression to adjust ITE ranks for PGY, sex, their interaction, and examination year, and we present estimated means and 95% CIs stratified by examination year and intervention status. All *P* values were 2 tailed, with significance set at *P* = .05. All analyses were conducted using Stata IC, version 15.1 (StataCorp LLC). Data analysis was conducted from October to December 2022.

## Results

### Participants

Of the 313 eligible residents, 216 completed the surveys. A total of 107 (49.5%) were female and 109 (50.5%) were male; 119 (55.1%) were PGY1 residents. Table 1 presents demographics, response rates, and unadjusted MBI-HSS(MP) scores per cohort. The response rates were 78.0% (85 of 109) for the preintervention cohort and 60.6% (63 of 104) and 68.0% (68 of 100) for the 2 postintervention cohorts. The PGY1 residents had higher response rates than PGY2 residents (119 of 152 [78.2%] vs 97 of 161 [60.2%]; *P* < .001). Men and women had similar response rates (108 of 155 [69.7%] vs 107 of 158 [67.7%]; *P* = .71).

**Table 1.** Eligible Participant and Respondent Characteristics and Unadjusted MBI-HSS(MP) Subscale Scores^a^

Characteristic	Total eligible (N = 313)	Academic year		
		2018-2019 (n = 109)^b^	2019-2020 (n = 104)^c^	2020-2021 (n = 100)^c^
Eligible participants, stratified by PGY				
PGY1	152 (48.6)	52 (47.7)	50 (48.1)	50 (50.0)
PGY2	161 (51.4)	57 (52.3)	54 (51.9)	50 (50.0)
Eligible participants, stratified by sex				
Male	155 (49.5)	63 (57.8)	49 (47.1)	43 (43.0)
Female	158 (51.4)	46 (42.2)	55 (52.9)	57 (57.0)
Other	0	0	0	0
Respondent PGY				
PGY1	119 (38.0)	45 (52.9)	36 (57.1)	38 (55.9)
PGY2	97 (31.0)	40 (47.1)	27 (42.9)	30 (44.1)
Missing	0	0	0	0
Respondent sex				
Male	108 (34.5)	50 (58.8)	28 (44.4)	30 (44.1)
Female	107 (34.2)	34 (40)	35 (55.6)	38 (55.9)
Other	0	0	0	0
Missing	1 (0.3)	1 (1.2)	0	0
Response rate				
Overall	216/313 (69.0)	85/109 (78.0)	63/104 (60.6)	68/100 (68.0)
PGY1	119/152 (78.2)	45/52 (86.5)	36/50 (72.0)	38/50 (76.0)
PGY2	97/161 (60.2)	40/57 (70.2)	27/54 (50.0)	30/50 (60.0)
Male	108/155 (69.7)	50/63 (79.4)	28/49 (57.1)	30/43 (69.8)
Female	107/158 (67.7)	34/46 (73.9)	35/55 (63.6)	38/57 (66.7)
Other	NA	0	0	0
Unadjusted MBI-HSS(MP) subscale score, median (IQR)				
Emotional exhaustion	NA	25 (19-30)	17 (10-22)	19.5 (13-25)
Depersonalization	NA	11 (8-15)	7 (3-10)	7 (3.5-11.5)
Personal achievement	NA	38 (33-41)	41 (36-43)	39 (36-42)

Abbreviations: MBI-HSS(MP), Maslach Burnout Inventory–Human Services Survey for Medical Personnel; NA, not applicable; PGY, postgraduate year.

^a^ Unless indicated otherwise, values are presented as No. (%) of respondents.

^b^ Preintervention cohort.

^c^ Postintervention cohorts.

### Outcomes

#### MBI-HSS(MP) Subcategories

**Figure 1.**
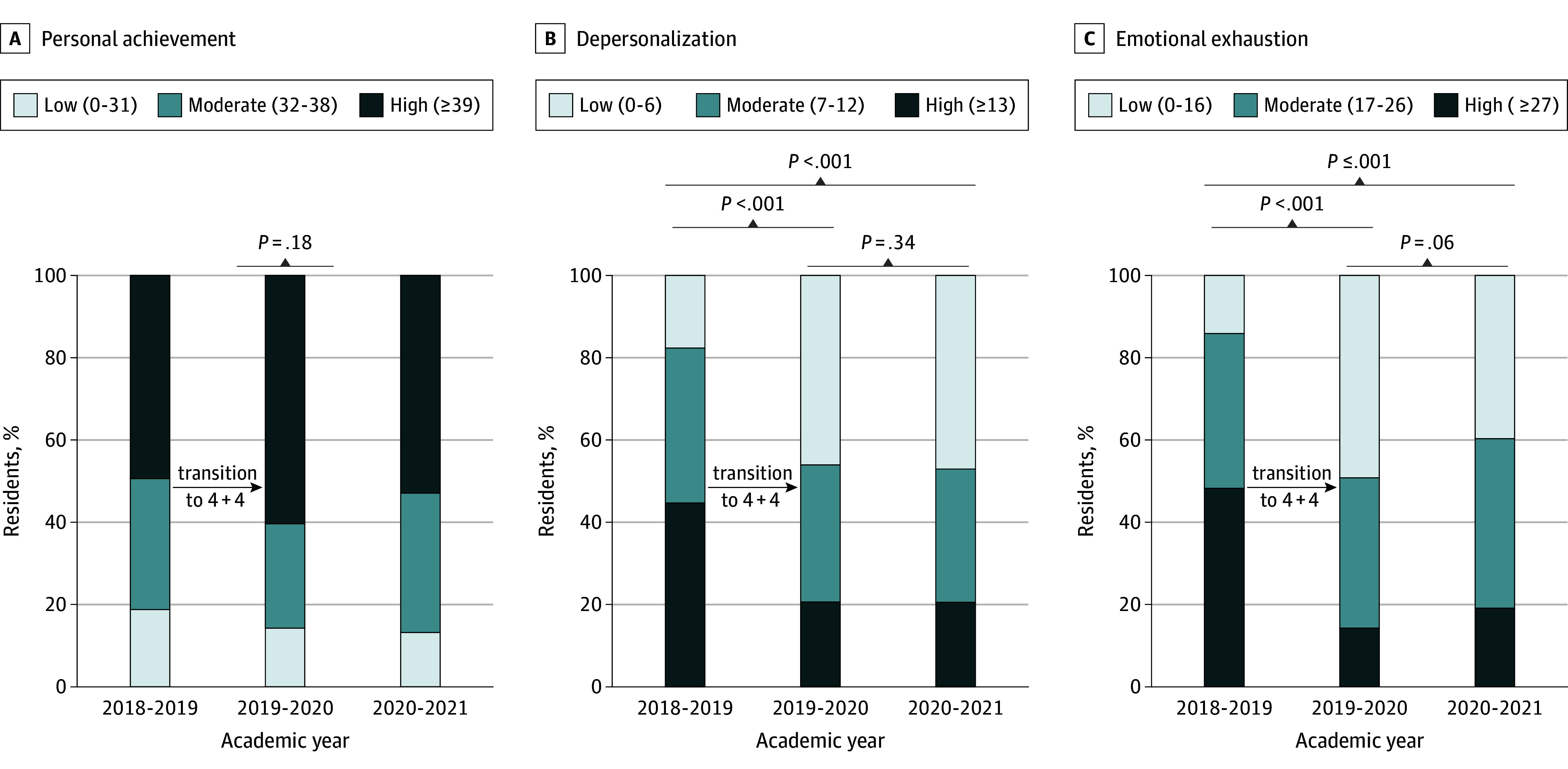
Preintervention and Postintervention Resident Survey Responses Regarding Perceived Impact of 4 + 4 Block Scheduling on Personal Achievement, Depersonalization, and Emotional Exhaustion Scores The Maslach Burnout Inventory–Human Services Survey for Medical Personnel was used to assess burnout in 3 subcategories: personal achievement (A), depersonalization (B), and emotional exhaustion (C). The 4 + 4 block schedule comprised 4-week inpatient call-based rotations and 4-week ambulatory non–call-based rotations. Scoring categories are defined in Lim et al.^22^

Adjusted EE scores (mean difference [MD], −6.78 [95% CI, −9.24 to −4.32]) and adjusted DP scores (MD, −3.81 [95% CI, −5.29 to −2.34]) were lower in the postintervention combined cohort. The change in PA scores did not reach statistical significance (MD, 1.4 [95% CI, −0.49 to 3.29]). Sensitivity analyses were consistent with these findings (Figure 1). High EE was prevalent among 48.2% of residents (41 of 85) before the intervention and only among 14.3% (9 of 63) and 19.1% (13 of 68) in the postintervention years (*P* < .001). High DP prevalence similarly decreased from 44.7% (38 of 85) to 20.6% (13 of 63) and remained unchanged at 20.6% (14 of 68) the last year (*P* < .001). Standardized mean differences indicated a moderate reduction in EE and DP (−0.74 [95% CI, −0.45 to −1.02] and −0.75 [95% CI, −0.47 to −1.03], respectively) and a slight increase in PA (0.20 [95% CI, −0.08 to 0.47]) (Table 2).

**Table 2.** Preintervention and Postintervention SMDs for Each Surveyed Outcome

Measured outcome	SMD (95% CI)^a^
MBI-HSS(MP) subcategory	
Emotional exhaustion	−0.74 (−0.45 to −1.02)
Depersonalization	−0.75 (−0.47 to −1.03)
Personal achievement	0.20 (−0.08 to 0.47)
Secondary outcomes survey	
Ability to acquire clinical skills	−0.23 (−0.50 to 0.05)
Ability to attend educational activities	1.11 (0.81 to 1.40)
Ability to participate in scholarly activity	2.10 (1.76 to 2.44)
Ability to acquire clinical reasoning skills	−0.21 (−0.49 to 0.06)
Availability of inpatient encounters	−0.70 (−0.97 to −0.41)
Availability of elective encounters	1.19 (0.89 to 1.48)
Availability of continuity clinic encounters	1.26 (0.96 to 1.55)
Professionalism	0.57 (0.29 to 0.85)
Job satisfaction	1.28 (0.98 to 1.58)
Morale	1.71 (1.39 to 2.03)
Time for activities outside of clinical setting	2.14 (1.80 to 2.14)
Satisfaction with career choice	1.19 (0.89 to 1.19)
Health	1.92 (1.58 to 2.24)
Time for friends and family	2.13 (1.79 to 2.13)
Overall well-being	2.08 (1.74 to 2.42)

Abbreviations: MBI-HSS(MP), Maslach Burnout Inventory–Human Services Survey for Medical Personnel; SMD, standardized mean difference.

^a^ The SMD is the difference in 2 sample means divided by the pooled SD (calculated using Cohen’s *D*); SMDs of 0.2, 0.5, and 0.8 indicate small, moderate, and large effect sizes, respectively. For emotional exhaustion and depersonalization subscales, a negative SMD indicates an improvement. For all other outcomes, a positive SMD indicates an improvement.

#### Secondary Outcomes

**Figure 2.**
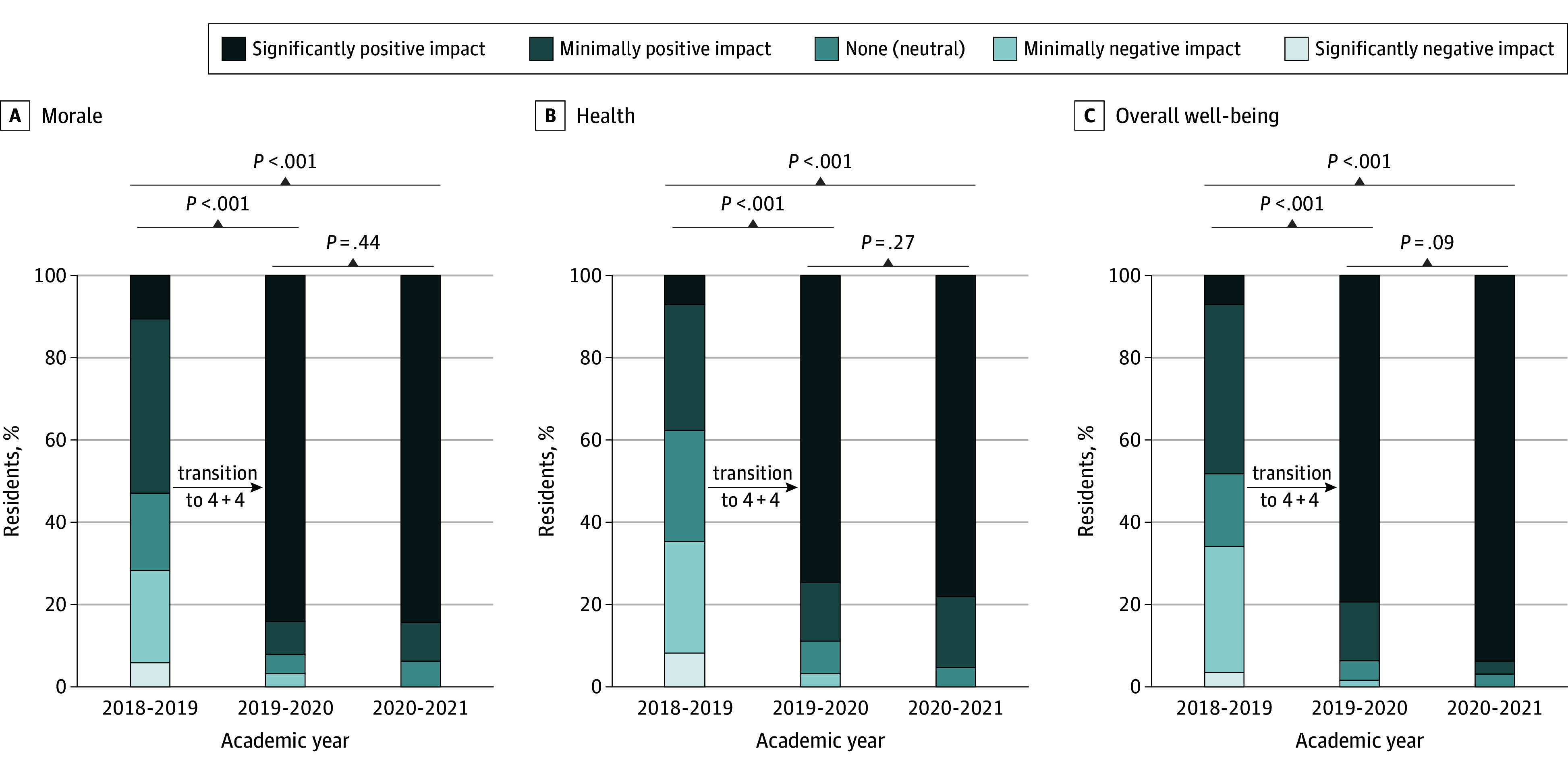
Preintervention and Postintervention Survey Responses Regarding Perceived Impact of 4 + 4 Block Scheduling on Morale, Health, and Well-Being A secondary outcomes survey was used to assess morale (A), health (B), and well-being (C). The 4 + 4 block schedule comprised 4-week inpatient call-based rotations and 4-week ambulatory non–call-based rotations.

The intervention was associated with a negative perception of inpatient encounter availability (significantly positive impact, 53 of 84 [63.1%] before the intervention to 15 of 63 [23.4%] to 24 of 64 [37.5%] after the intervention; *P* < .001). In terms of perceived ability to acquire clinical skills and reasoning, no statistically significant differences were found (questions 1 and 4; eTable 1 in Supplement 1). Responses to the remaining 12 questions reached statistical significance, noting improvement in the ability to participate in scholarly activities, availability of continuity clinic encounters, job satisfaction, and overall well-being, among others (eTable 1 in Supplement 1). For example, for morale, health, and well-being, the number of respondents entering a significantly positive response increased from 7 of 100 (7.0%) to 11 of 100 (11.0%) before the intervention to 75 of 100 (75.0%) to 84 of 100 (84.0%) after the intervention (Figure 2). Standardized mean differences indicated a large positive association for 11 items in secondary outcomes (SMDs >1.0), especially for time for scholarly activities, experiences outside of the clinical setting, time with family and friends, and overall well-being, with SMDs exceeding 2.0 (Table 2). The only negative association noted by the respondents was the availability of inpatient encounters (SMD, 0.69 [95% CI, −0.97 to −0.41]).

#### ITE Scores

All variations of the analyses of preintervention and postintervention ITE percentile ranks (adjusted and unadjusted) showed similar results (Table 3 and eFigure and eTables 2-4 in Supplement 1). Differences in ITE percentile ranks before and after the intervention did not reach statistical significance.

**Table 3.** Adjusted In-Training Service Examination Percentile Ranks

	Academic year						*P* value
	2017-2018	2018-2019	2019-2020^a^	2020-2021	2021-2022	2022-2023	
No. of participants	108	108	104	100	65	103	NA
Mean percentile rank (95% CI)	72.3 (68.6-76.0)	71.0 (67.2-74.7)	74.0 (70.2-77.8)	74.8 (70.9-78.7)	71.4 (66.6-76.3)	74.1 (70.2-77.8)	.68

Abbreviation: NA, not applicable.

^a^ The first year with the 4 + 4 schedule (4-week inpatient call-based rotations and 4-week ambulatory non–call-based rotations).

## Discussion

To our knowledge, this study is the first to suggest a positive association between a 4 + 4 block training schedule and internal medicine resident burnout scores. The preintervention prevalence of high EE and DP scores (48.2% and 44.7%, respectively) was similar to that of other US and Canadian internal medicine residencies.^5,23,28^ The results of this study suggest that the transition to a 4 + 4 schedule was associated with markedly improved EE and DP domains of burnout to a degree not previously documented with any other intervention.^14^ For instance, prior studies evaluating work-hour reductions demonstrated a mean decrease in EE and DP scores by 2.7 and 1.7 points, respectively,^14^ whereas our intervention was associated with EE and DP score reductions of 6.8 and 3.8 points, respectively. Other prior efforts to reduce resident burnout, including self-care workshops and meditation, have resulted in more modest improvements.^14^ The magnitude of change carries significance because in prior studies of physicians, a 1-point increase in the EE subscale score has been associated with a 7% increase in suicidal ideation and a 5% to 6% increase in major medical errors.^3,29^

Our 4 + 4 training schedule was associated with resident reports of a positive impact on perceived aspects of professional development, including morale, job satisfaction, professionalism, ability to attend educational activities, and ability to participate in scholarly activity. The postintervention resident cohorts noted improved perceived wellness, including time for friends and family, activities outside of medicine, and overall health and well-being. Although the preintervention responses were similar to those of the iCOMPARE study participants, our postintervention cohorts reported vastly higher satisfaction with the rotation schedule.^23^ Additionally, there was no detriment in the perceived ability to acquire clinical skills or develop clinical reasoning. In this study, residents’ only perceived negative impact of the intervention was on the availability of inpatient clinical opportunities. Reassuringly, there was no change in medical knowledge across multiple years, as demonstrated by ITE scores. Although many programs have transitioned to X + Y scheduling, there is a paucity of data surrounding the implications of these schedules in terms of resident burnout, clinical competency, and educational experience.^21,30^ Our study is, to our knowledge, the first to suggest that a specific X + Y schedule (4 + 4) may have a positive association across multiple domains.

Improving resident physician burnout, quality of life, and professional identity formation may have far-ranging implications beyond wellness. Resident and attending physician burnout may adversely affect patient care.^2,4,6^ Additionally, burnout can negatively affect physician engagement, resulting in reduced professional identity formation and higher turnover rates.^4,31^ Our 4 + 4 schedule was associated with a sustained improvement in burnout over a 2-year period, even during a global pandemic.

Multiple features of the 4 + 4 schedule may contribute to better resident outcomes. First, although we did not directly measure patient-care hours or total hours worked, there was a redistribution of total patient-care hours favoring outpatient care and more structured professional time dedicated to scholarly activity and other aspects of professional development. Although reductions in total work hours have not been previously demonstrated to improve burnout,^32^ our schedule structure provided more opportunity for other processes previously determined to prevent burnout, including reduced overall clinical constraints.^33^ Second, the intervention schedule allowed for more autonomy in selection of activities outside of clinical time, facilitating more longitudinal and personal connections to be made, which has been shown to reduce burnout.^28^ Further, the structure increased the interval between rotations with higher rates of moral distress.^5^ Finally, the 4 + 4 schedule findings encompassed 4 of 5 core themes identified as contributing to or preventing burnout^34^ as follows: (1) having or lacking a sense of meaning at work: postintervention cohorts scored higher in self-perceived professionalism, job satisfaction, morale, and satisfaction with career choice; (2) fatigue and exhaustion: residents reported improved health and overall well-being; (3) the steep learning curve of residency: by smoothing the curve of inpatient opportunities by spacing them at intervals, residents reported improved ability to attend educational sessions without decrement in clinical reasoning or acquisition of clinical skills; and (4) social relationships at and outside work: residents felt they had more time for friends and family and activities outside of work.

### Limitations

Our study has several limitations. First, our survey response rate dropped from 78.0% to 60.6% and 68.0% in the 2 postintervention years, raising concern for nonresponse bias. The transition from in-person (before the intervention) to electronic (after the intervention, due to the global pandemic) survey administration may have affected the response rates, as electronic delivery of surveys has been demonstrated to yield lower response rates.^35,36^ Additionally, recent studies have suggested that the association between response rates and nonresponse bias may be weaker than previously thought.^37,38^ Second, our postintervention cohorts spanned the COVID-19 pandemic, which may have introduced additional confounding in ways that are difficult to ascertain.^7,39,40^ Due to pandemic-induced staffing needs and reduction in research and scholarly activities at our institutions, there were a variety of minor differences to our schedule during the 2 postintervention years. Third, the second postintervention cohort participants had no prior experience with the 4 + 1 schedule, which could have factored into their responses. Reassuringly, we did not observe marked differences in any of the MBI-HSS(MP) subscales or other secondary outcomes between the 2 postintervention cohorts. Fourth, the survey was anonymized, and we cannot directly compare individual results. Although there were no differences in the response rates stratified by sex, we did observe higher response rates by the PGY1 residents, which could have affected the results. The adjusted analysis that included PGY status and sex as potential confounders was consistent with the unadjusted analysis. Fifth, other cointerventions, including a novel well-being curriculum for interns (5 hours of self-reflection and small group discussion) and minor structural IMRP changes between the preintervention and postintervention periods, may have mediated the results. Finally, the first postintervention cohort (2020) was not fully independent from the preintervention cohort, which may have affected the output of the statistical tests that assume sample independence. Although anonymous survey collection complicated the statistical analysis, the potential tradeoff was creation of psychological safety for residents to maximize accurate reflection and survey participation. The observed changes persisted in the fully independent postintervention cohort (2021), providing some reassurance that sample overlap was not a major factor in the overall findings.

## Conclusions

The findings of this survey study of internal medicine resident physicians suggest that 4 + 4 block scheduling for an IMRP, compared with a 4 + 1 schedule, may be associated with a substantial improvement in internal medicine resident burnout. Furthermore, residents reported markedly improved health, wellness, professional development, and engagement of trainees without a decrement in perceived clinical skills or standardized examination scores. These results suggest that specific X + Y block combinations may be better than others and warrant consideration for investigation and adoption by other IMRPs nationwide. Further study is needed to determine whether these results persist after graduation from residency.

## Notes

Supplement 1. **eAppendix 1.** Rotation Schedule Description **eTable 1.** Secondary Outcomes **eTable 2.** Adjusted In-Training Service Examination Scores, Dichotomized by Transition to a New Rotation Structure **eTable 3.** Unadjusted In-Training Service Examination Scores **eTable 4.** Unadjusted In-Training Service Examination Scores, Dichotomized by Transition to a New Rotation Structure **eFigure.** Predicted Mean In-Training Examination Percentile Ranks, Adjusted for Sex, Postgraduate Year Level, and Examination Year **eAppendix 2.** Educational Outcomes and Impact on Trainee Burnout of an Innovative Training Schedule and Curricular Structure in Internal Medicine Residency Training (4 + 4): Survey Questionnaire

Supplement 2.
Data Sharing Statement
