# Supplementary material for: Resident Burnout, Wellness, Professional Development, and Engagement Before and After New Training Schedule Implementation
Source: JAMA Netw Open. 2024 Feb 28;7(2):e240037. doi: 10.1001/jamanetworkopen.2024.0037 (PMC10902722; doi:10.1001/jamanetworkopen.2024.0037)
Supplement: Supplement 1. — eAppendix 1. Rotation Schedule Description eTable 1. Secondary Outcomes eTable 2. Adjusted In-Training Service Examination Scores, Dichotomized by Transition to a New Rotation Structure eTable 3. Unadjusted In-Training Service Examination Scores eTable 4. Unadjusted In-Training Service Examination Scores, Dichotomized by Transition to a New Rotation Structure eFigure. Predicted Mean In-Training Examination Percentile Ranks, Adjusted for Sex, Postgraduate Year Level, and Examination Year eAppendix 2. Educational Outcomes and Impact on Trainee Burnout of an Innovative Training Schedule and Curricular Structure in Internal Medicine Residency Training (4 + 4): Survey Questionnaire [file jamanetwopen-e240037-s001.pdf]

Supplementary Online Content

Heppe D, Baduashvili A, Limes JE, et al. Resident burnout, wellness, professional development, and engagement before and after new training schedule implementation. *JAMA Netw Open*. 2024;7(2):e240037. doi:10.1001/jamanetworkopen.2024.0037

eAppendix 1. Rotation Schedule Description

eTable 1. Secondary Outcomes

eTable 2. Adjusted In-Training Service Examination Scores, Dichotomized by Transition to a New Rotation Structure

eTable 3. Unadjusted In-Training Service Examination Scores

eTable 4. Unadjusted In-Training Service Examination Scores, Dichotomized by Transition to a New Rotation Structure

eFigure. Predicted Mean In-Training Examination Percentile Ranks, Adjusted for Sex, Postgraduate Year Level, and Examination Year

eAppendix 2. Educational Outcomes and Impact on Trainee Burnout of an Innovative Training Schedule and Curricular Structure in Internal Medicine Residency Training (4+4): Survey Questionnaire

This supplementary material has been provided by the authors to give readers additional information about their work.

© 2024 Heppe D et al. *JAMA Network Open.*

eAppendix 1. Rotation Schedule Description

In the 4+4 model, residents participate in a total of six inpatient based rotations and six ambulatory based rotations each year of training for all three years of residency. In a given academic year, three of the ambulatory based rotations are continuity clinic and the remaining three are elective rotations. Inpatient blocks alternate with outpatient blocks, ensuring residents do not have a call-based rotation for more than 4 weeks at a time and that the intervening outpatient time is 4 weeks.

There are 12 four week rotations, accounting for 48 weeks of 52 weeks. We divided the remaining weeks of the year as 2 basecamp weeks at the beginning of the academic year to train interns for clinic and ward readiness. For 2 weeks during the December-January holidays we close all electives. Half the residency program work in the hospital to cover inpatient services for 1 week then take vacation the second week or vice versa. This allows residents to take 1 holiday week off during a popular time of the year to be on vacation.

A resident's weekly schedule while on continuity clinic rotation consists of 10 half day sessions allocated as follows: 4 continuity clinic direct patient care, 1 task management indirect patient care, 1 education, 1 subspecialty clinic, 1 quality improvement/clinic site specific education, 1 research/scholarly activity, and 1 personal development session. This is in contrast to our prior model (4+1) in which trainees spent 4 weeks on a dedicated inpatient rotation or outpatient elective followed by one full week of primary care continuity clinic. Two half days per continuity clinic week (occurring once every 5 weeks) were dedicated to educational activities and curriculum specific to medical education, leadership, and research. Residents had no protected time for EHR in basket management, scholarly pursuits, or personal development in our prior model.

Year Overview in 4+4 Block Scheduling

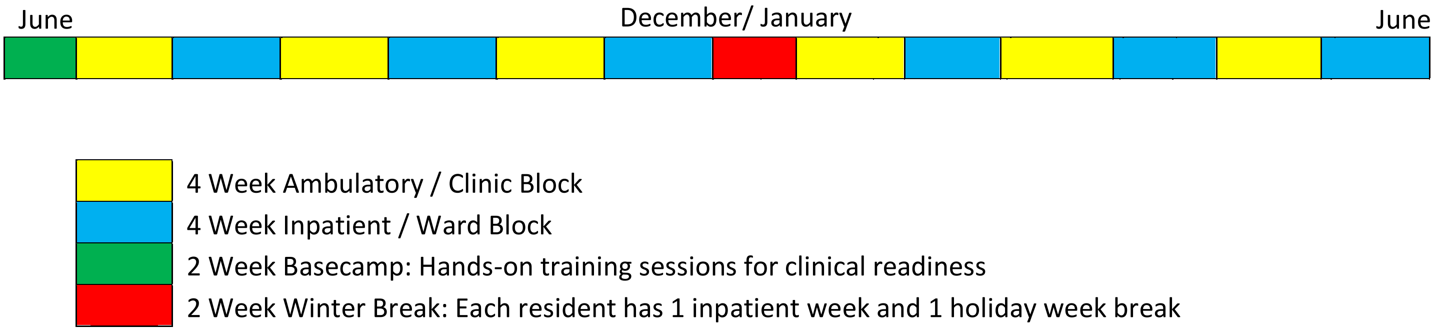

Sample Continuity Clinic Week

	Mon	***Tue***	***Wed***	***Thu***	***Fri***
AM	Clinic Didactics/Quality Improvement	Scholarly Activity/Research	Education	Clinic	Clinic
PM	Clinic	Clinic	Task Management	Subspecialty Clinic	Personal Development

© 2024 Heppe D et al. *JAMA Network Open.*

eTable 1. Secondary Outcomes

	Likert Scale (%)[a]					*P* value for overall difference[b]	Pairwise comparison *P* values[c]		
**Secondary outcome questions**	1	2	3	4	5		AY18-19 to AY19-20	AY18-19 to AY20-21	AY19-20 to AY20-21
Ability to acquire clinical skills						0.04			
AY 2018-19	0	5	21	24	51				
AY 2019-20	2	10	30	25	33				
AY 2020-21	0	6	20	25	48				
Ability to attend educational activities						<0.001	<0.001	<0.001	0.21
AY 2018-19	0	25	24	40	12				
AY 2019-20	0	3	17	24	56				
AY 2020-21	0	2	14	22	63				
Ability to participate in scholarly activity						<0.001	<0.001	<0.001	0.37
AY 2018-19	15	33	21	22	8				
AY 2019-20	0	2	10	13	76				
AY 2020-21	0	0	6	16	78				
Ability to acquire clinical reasoning skills						0.14			
AY 2018-19	0	0	29	22	48				
AY 2019-20	0	10	32	22	37				
AY 2020-21	0	3	25	28	44				
Availability of inpatient encounters						<0.001	<0.001	0.004	0.006
AY 2018-19	0	5	18	14	63				
AY 2019-20	3	16	44	13	24				
AY 2020-21	2	6	31	23	38				
Availability of elective encounters						<0.001	<0.001	<0.001	0.06
AY 2018-19	11	27	24	26	13				
AY 2019-20	2	3	24	22	49				

© 2024 Heppe D et al. *JAMA Network Open.*

AY 2020-21	0	3	14	19	64				
Availability of continuity clinic encounters						<0.001	<0.001	<0.001	0.05
AY 2018-19	9	21	24	31	15				
AY 2019-20	0	3	14	30	52				
AY 2020-21	0	0	14	14	72				
Professionalism						<0.001	0.01	<0.001	0.02
AY 2018-19	0	4	65	11	21				
AY 2019-20	0	0	49	19	32				
AY 2020-21	0	0	34	13	53				
Job satisfaction						<0.001	<0.001	<0.001	0.36
AY 2018-19	2	16	19	45	18				
AY 2019-20	0	2	6	16	76				
AY 2020-21	0	2	9	8	81				
Morale						<0.001	<0.001	<0.001	0.44
AY 2018-19	6	22	19	42	11				
AY 2019-20	0	3	5	8	84				
AY 2020-21	0	0	6	9	84				
Time for activities outside of clinical setting						<0.001	<0.001	<0.001	0.24
AY 2018-19	15	28	12	36	8				
AY 2019-20	0	2	6	3	89				
AY 2020-21	0	0	3	2	95				
Satisfaction with career choice						<0.001	<0.001	<0.001	0.24
AY 2018-19	0	7	47	26	20				
AY 2019-20	0	0	16	19	65				
AY 2020-21	0	0	14	13	73				
Health						<0.001	<0.001	<0.001	0.27
AY 2018-19	8	27	27	31	7				
AY 2019-20	0	3	8	14	75				

© 2024 Heppe D et al. *JAMA Network Open.*

AY 2020-21	0	0	5	17	78				
Time for friends and family						<0.001	<0.001	<0.001	0.15
AY 2018-19	18	27	9	39	7				
AY 2019-20	0	2	5	10	84				
AY 2020-21	0	0	3	2	95				
Overall well-being						<0.001	<0.001	<0.001	0.09
AY 2018-19	4	31	18	41	7				
AY 2019-20	0	2	5	14	79				
AY 2020-21	0	0	3	3	94				

AY=academic year

a – the Likert Scale was structured as follows: (1) significantly negative impact, (2) minimally negative impact, (3) neutral impact, (4) minimally positive impact, (5) significantly positive impact

b – obtained using Kruskal-Wallis test

c – obtained using Dunn test

eTable 2. Adjusted In-Training Service Examination Scores, Dichotomized by Transition to a New Rotation Structure

	Pre-transition (AY 2017-2019)	Post-transition (AY 2020-2023)	*P* value
Participants (n)	216	372	
Adjusted Mean %ile rank (95% CI)	71.6 (69.0 – 74.2)	73.7 (71.8-75.8)	0.11

AY=academic years

© 2024 Heppe D et al. *JAMA Network Open.*

**eTable 3.** Unadjusted In-Training Service Examination Scores							
	Academic Year						
	2017-18	2018-19	2019-20[a]	2020-21	2021-22	2022-23	*P* value
Participants (n)	108	108	104	100	65	103	
Mean %ile rank (SD)	72.7 (19.3)	71.3 (21.4)	73.3 (19.6)	74.6 (20.7)	72.2 (18.9)	73.7 (18.9)	0.88[b]
Median %ile rank (IQR)	77 (60 – 90)	76 (56 – 90)	77 (63.5 – 90)	79.5 (61 – 91.5)	76 (57 – 88)	75 (61 – 89)	0.82[c]

SD=standard deviation, IQR=interquartile range

a – First year with the 4+4 schedule

b – *P* value obtained using one-way ANOVA. Bartlett's test for equal variance – *P* value=0.765

c – *P* value obtained using Kruskal Wallis test adjusted for ties

**eTable 4.** Unadjusted In-Training Service Examination Scores, Dichotomized by Transition to a New Rotation Structure			
	Pre-transition (AY 2017-2019)	Post-transition (AY 2020-2023)	*P* value
Participants (n)	216	372	
Mean %ile rank (SD)	72.0 (20.3)	73.6 (19.5)	0.83[a]
Median %ile rank (IQR)	76 (59 – 90)	77.5 (61 – 90)	0.40[b]

AY=academic years, SD=standard deviation, IQR=interquartile range

a – *P* value obtained using two-sample t-test with equal variances

b – *P* value obtained using two-sample Wilcoxon Rank-sum test

© 2024 Heppe D et al. *JAMA Network Open.*

eFigure. Predicted Mean ITS Percentile Ranks, Adjusted for Sex, Postgraduate Year Level, and Examination Year

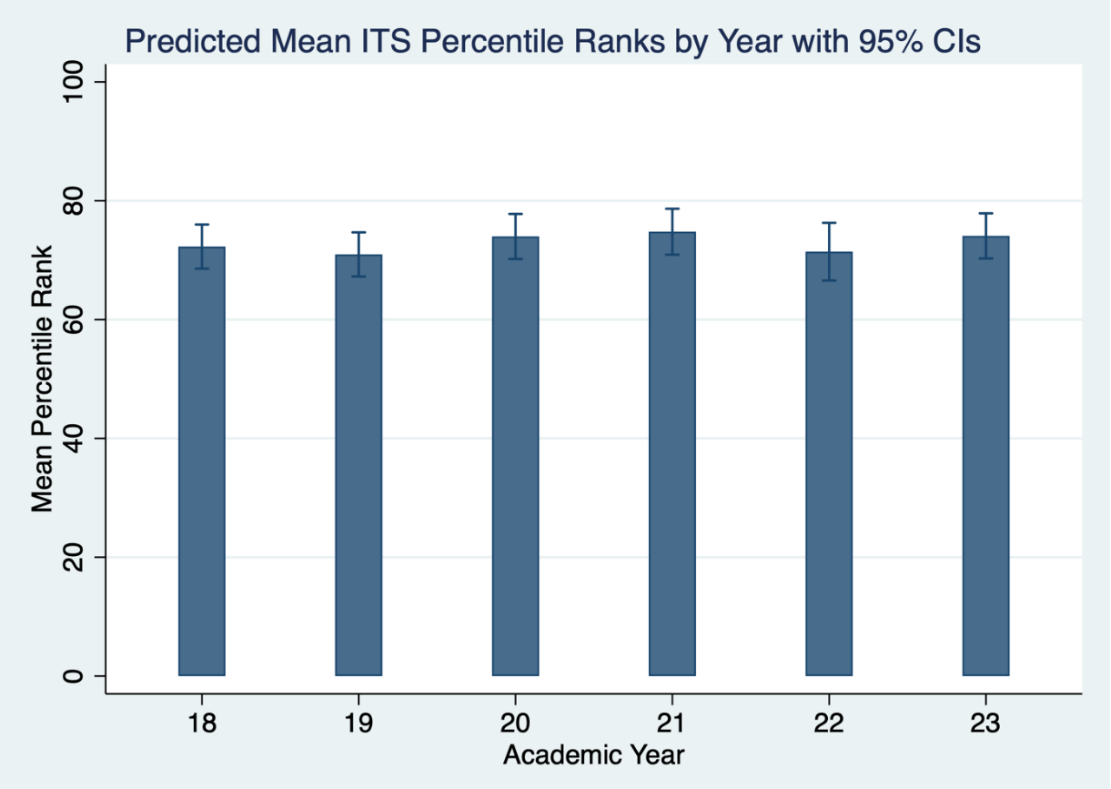

© 2024 Heppe D et al. *JAMA Network Open.*

eAppendix 2. Educational Outcomes and Impact on Trainee Burnout of an Innovative Training Schedule and Curricular Structure in Internal Medicine Residency Training (4+4): Survey Questionnaire

Dear Residents,

We are initiating a significant change in the schedule and curricular structure of our internal medicine residency training program. We are interested in ensuring that it is a successful and positive change, particularly in relation to educational outcomes and quality of life of the residents.

As such, you are invited to participate in a research study examining some of the educational outcomes, quality of life and burnout in residents participating in the new training structure compared with the prior. All catagorical/primary care and hospitalist interns and second year residents at the UC Internal Medicine Residency Program are eligible to participate in the study. Participation in the study includes completion of a brief paper survey. We will assume that you are willing participant if you submit your answers. Do not include any identifying on the survey. We will not attempt to identify anyone from their demographic information.

There are no known risks to participating in this study. We will not attempt to identify subjects via their responses. No names will appear on or be used on research documents or be used in presentations or publications.

We hope to receive completed surveys/questionnaires from all surveyed residents. Please note you have a choice about whether to complete the survey/questionnaire. If you do participate, you are free to skip any questions or discontinue participation at any time. Although you will not personally benefit from taking part in this research study, your responses will contribute to a greater understanding of the efficacy of our new schedule and curricular structure

The survey/questionnaire will take about 15 minutes to complete. If you have questions about the study, please feel free to contact the authors whose contact information is given below Thanks so much,

Geoff Connors, Dan Heppe, Julia Limes, Karen Chacko, Emily Gottenberg, Katie Suddarth, Lisa Davis, Mark Kearns.

© 2024 Heppe D et al. *JAMA Network Open.*

2019 Survey
Group Characteristics:
Training Level:
a) PGY1
b) PGY2
Gender
a) Female
b) Male
c) Other
What category of rotation did you most recently complete: a) Inpatient Rotation
b) Outpatient or Elective Rotation

Section 1: Impact of 4+4 in comparison to prior 4+1
Instructions: Please take into consideration the impact of your most recent year of internal medicine

residency training when answering the following questions:

1. Ability to acquire clinical skills

a. Significant positive impact b. Minimal positive impact
c. No impact (neutral)
d. Minimal negative impact
e. Significant negative impact.

2. Ability to attend educational activities

a. Significant positive impact b. Minimal positive impact
c. No impact (neutral)
d. Minimal negative impact
e. Significant negative impact.

3. Ability to participate in scholarly activity

a. Significant positive impact b. Minimal positive impact
c. No impact (neutral)
d. Minimal negative impact
e. Significant negative impact.

4. Ability to acquire clinical reasoning skills

a. Significant positive impact b. Minimal positive impact
c. No impact (neutral)
d. Minimal negative impact
e. Significant negative impact.

5. Availability of inpatient encounters

© 2024 Heppe D et al. *JAMA Network Open.*

a. Significant positive impact b. Minimal positive impact
c. No impact (neutral)
d. Minimal negative impact
e. Significant negative impact.

6. Availability of elective encounters

a. Significant positive impact b. Minimal positive impact
c. No impact (neutral)
d. Minimal negative impact
e. Significant negative impact.

7. Availability of continuity clinic encounters

a. Significant positive impact b. Minimal positive impact
c. No impact (neutral)
d. Minimal negative impact
e. Significant negative impact.

8. Professionalism

a. Significant positive impact b. Minimal positive impact
c. No impact (neutral)
d. Minimal negative impact
e. Significant negative impact.

9. Job Satisfaction

a. Significant positive impact b. Minimal positive impact
c. No impact (neutral)
d. Minimal negative impact
e. Significant negative impact.

10. Morale

a. Significant positive impact b. Minimal positive impact
c. No impact (neutral)
d. Minimal negative impact
e. Significant negative impact.

11. Time for activities outside of clinical setting

a. Significant positive impact b. Minimal positive impact
c. No impact (neutral)
d. Minimal negative impact
e. Significant negative impact.

© 2024 Heppe D et al. *JAMA Network Open.*

12. Satisfaction with career choice

a. Significant positive impact b. Minimal positive impact
c. No impact (neutral)
d. Minimal negative impact
e. Significant negative impact.

13. Health

a. Significant positive impact b. Minimal positive impact
c. No impact (neutral)
d. Minimal negative impact
e. Significant negative impact.

14. Time for friends and family

a. Significant positive impact b. Minimal positive impact
c. No impact (neutral)
d. Minimal negative impact
e. Significant negative impact.

15. Overall well being

a. Significant positive impact b. Minimal positive impact
c. No impact (neutral)
d. Minimal negative impact
e. Significant negative impact.

Section 2: Maslach Burnout Inventory:

Instructions: Please take into consideration your most recent year of internal medicine residency training when answering this questionnaire. See Separate Questionaire.

© 2024 Heppe D et al. *JAMA Network Open.*
