# Supplementary material for: Resident Burnout, Wellness, Professional Development, and Engagement Before and After New Training Schedule Implementation
Source: JAMA Netw Open. 2024 Feb 28;7(2):e240037. doi: 10.1001/jamanetworkopen.2024.0037 (PMC10902722; doi:10.1001/jamanetworkopen.2024.0037)
Supplement: Supplement 2. — Data Sharing Statement [file jamanetwopen-e240037-s002.pdf]

Data Sharing Statement

Heppe. Resident Burnout, Wellness, Professional Development, and Engagement Before and After New Training Schedule Implementation. *JAMA Netw Open*. Published February 28, 2024. doi:10.1001/jamanetworkopen.2024.0037

Data
Data available: No
